# Combining Monte Carlo methods with coherent wave optics for the simulation of phase-sensitive X-ray imaging

**DOI:** 10.1107/S1600577514000952

**Published:** 2014-03-18

**Authors:** Silvia Peter, Peter Modregger, Michael K. Fix, Werner Volken, Daniel Frei, Peter Manser, Marco Stampanoni

**Affiliations:** a Swiss Light Source, Paul Scherrer Institut, CH-5232 Villigen, Switzerland; b Institute for Biomedical Engineering, Eidgenössische Technische Hochschule Zürich, CH-8092 Zürich, Switzerland; c Centre d’Imagerie BioMedicale, Ecole Polytechnique Federale de Lausanne, CH-1015 Lausanne, Switzerland; d Division of Medical Radiation Physics and Department of Radiation Oncology, Inselspital, University Hospital Bern and University of Bern, CH-3010 Bern, Switzerland

**Keywords:** X-ray phase-contrast imaging, grating interferometer, Monte Carlo simulations

## Abstract

Phase-sensitive X-ray imaging shows a high sensitivity towards electron density variations, making it well suited for imaging of soft tissue matter. However, there are still open questions about the details of the image formation process. Here, a framework for numerical simulations of phase-sensitive X-ray imaging is presented, which takes both particle- and wave-like properties of X-rays into consideration. A split approach is presented where we combine a Monte Carlo method (MC) based sample part with a wave optics simulation based propagation part, leading to a framework that takes both particle- and wave-like properties into account. The framework can be adapted to different phase-sensitive imaging methods and has been validated through comparisons with experiments for grating interferometry and propagation-based imaging. The validation of the framework shows that the combination of wave optics and MC has been successfully implemented and yields good agreement between measurements and simulations. This demonstrates that the physical processes relevant for developing a deeper understanding of scattering in the context of phase-sensitive imaging are modelled in a sufficiently accurate manner. The framework can be used for the simulation of phase-sensitive X-ray imaging, for instance for the simulation of grating interferometry or propagation-based imaging.

## Abstract 2

A framework combining wave-optics with Monte Carlo methods for numerical simulations of phase-sensitive X-ray imaging has been developed.

## 1. Introduction   

In recent years, a wide variety of techniques for phase-sensitive X-ray imaging have been developed. Crystal interferometry (Bonse & Hart, 1965 ▶; Momose *et al.*, 1996 ▶) has a high sensitivity to phase variations, but is limited with respect to field of view. Analyser-based imaging (Davis *et al.*, 1995 ▶; Stampanoni *et al.*, 2002 ▶; Modregger *et al.*, 2007 ▶) has a larger field of view, but requires a monochromatic beam. Phase propagation imaging (Cloetens *et al.*, 1996 ▶; Snigirev *et al.*, 1995 ▶) offers the advantage of a comparably simple experimental set-up and the possibility to acquire high-resolution images at high speed. A phase-sensitive imaging technique which exploits absorption, phase and dark-field contrast is grating-based hard X-ray interferometry (GI) (Momose *et al.*, 2003 ▶; David *et al.*, 2002 ▶). GI has been shown to have a particularly high sensitivity to electron density variations, making it well suited for biological imaging (McDonald *et al.*, 2009 ▶; Qi *et al.*, 2010 ▶). An additional advantage of GI is the comparatively low coherence requirement, which allows not only the use of synchrotron sources but, utilizing a third grating, also of standard laboratory X-ray tubes (Pfeiffer *et al.*, 2006 ▶). This makes GI of special interest for medical applications and in recent years a lot of effort has been put into making this technique available for clinical applications (Stampanoni *et al.*, 2011 ▶; Tang *et al.*, 2012 ▶; Li *et al.*, 2011 ▶).

The particle–wave duality of photons suggests two distinctive ways to consider X-rays, which offer complementary insight into the interactions of X-rays with matter. On the one hand, the particle character is well suited to describe effects such as Compton or Rayleigh scattering or photoelectric absorption. On the other hand, the wave character offers a convenient way to describe coherent effects such as phase shifts. The different interactions of X-rays with matter lead to different kinds of contrasts in X-ray imaging. A grating interferometer delivers three kinds of contrast (absorption, differential phase and dark-field), corresponding to three different X-ray interaction processes. Specifically, the dark-field contrast is related to scattering and phase contrast relies on beam coherence. This indicates that for realistic investigations of the image formation process it is essential that both particle- and wave-like properties of X-rays are taken into account.

We present a simulation framework that takes both behaviours in that sense into account by combining wave optics with Monte Carlo methods (MC). Wave optics simulations treat X-rays as an electromagnetic wave, which opens the possibility to model interference (Weitkamp, 2004 ▶). In MC the path of individual particles through matter is modelled based on probabilities of scattering and absorption obtained from the physical cross sections of the material. MC methods are widely used for the deep investigation of X-ray imaging techniques, particularly for dose estimations (in a computed tomography scanner for instance) or scattering corrections in cone-beam computed tomography systems. Through the combination of wave optics and MC, absorption, phase-shift, interference and scattering can be modelled within one framework. Recently a number of publications have been made that investigate the dark-field contrast formation mechanism (Bech *et al.*, 2010 ▶; Yashiro *et al.*, 2010 ▶; Wang *et al.*, 2009*b* ▶; Jensen *et al.*, 2010 ▶; Lynch *et al.*, 2011 ▶; Chen *et al.*, 2010 ▶) and different efforts to investigate GI using MC have already been published (Cong *et al.*, 2012 ▶; Bartl *et al.*, 2010 ▶), but, so far in the investigation of X-ray scattering, the particle and wave approach have been considered separately. However, our approach should provide new insights into the matter, since it combines MC with wave optics within the same framework. This should provide an ideal tool for the investigation of the scattering process within a sample that can for instance be used to investigate the physical interpretation as well as the potential application of the ultrasmall-angle X-ray scattering with GI (Modregger *et al.*, 2012 ▶). Owing to the combination of MC and wave optics, our framework can be used for different phase-sensitive X-ray imaging methods; it would also be applicable for the exploration of the parameter space or optimization of different set-ups. An additional advantage of using MC is the possibility to calculate dose distributions, which is of interest for future medical applications of phase-sensitive X-ray imaging.

## 2. Materials and methods   

### 2.1. The simulation framework   

**Figure 1:**
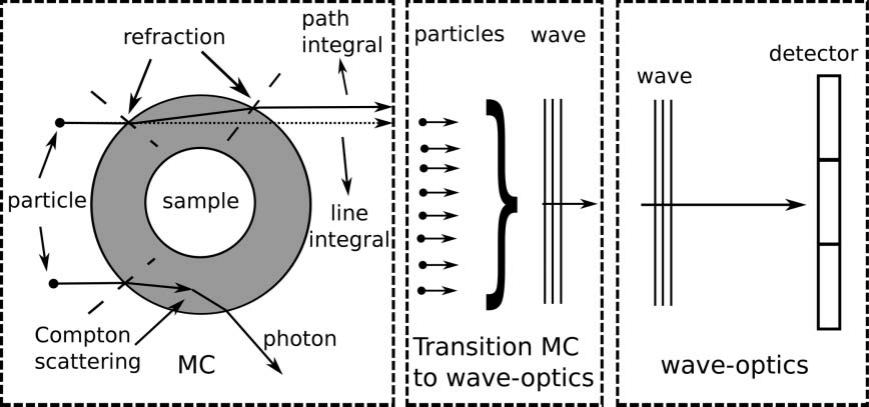
Sketch of the simulation framework, which is divided into three parts. On the left-hand side there is the sample part which is implemented in MC, and on the right the imaging part which is implemented using wave optics. In between there is the transition from MC to wave optics that is described in §2.1.2.

Phase-shift and absorption of a material are described by the real part δ and imaginary part β of its refractive index *n* = 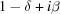. β is related to the attenuation coefficient μ through μ = 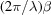, where λ is the wavelength. The attenuation coefficient of a material consisting of only one element with atomic mass *A* is related to the total cross section 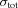 through (Hubbell, 1997 ▶)
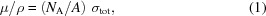
where ρ denotes the mass density of the material and 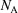 is Avogadro’s number. The total cross section 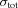 can be written as the sum of the cross sections of contributing photon interactions in the material. In the considered X-ray energy range, between 1 and 25 keV, the relevant contributions to the cross sections are due to the photoelectric absorption 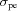, Compton scattering 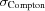 and Rayleigh scattering 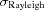. The total cross section can thus be written as
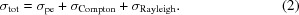
In the case of a material consisting of different elements, the mass attenuation coefficient 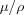 is the weighted sum over the individual mass attenuation coefficients 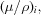


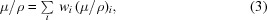
where 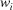 denotes the fraction of element *i* per weight. The real decrement δ for a wavelength λ is connected to the composition of a material through (James, 1954 ▶)
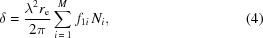
where the sum runs over all elements within the material, 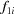 is the real part of the atomic scattering factor of element *i*, 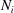 stands for the number of atoms of element *i* per unit volume, and *r*_e_ = 2.82 × 10^−15^ m denotes the classical electron radius. For energies sufficiently far away from an absorption edge, the scattering factor is approximately equal to the atomic number, 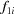 ≃ 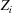 (Henke *et al.*, 1993 ▶). Since the scattering processes, which are relevant for the contrast formation, take place in the sample and the interference occurs in front of the detector, the simulation package was split into two parts: the MC-based sample part and the wave-optics-based propagation part. In between the two parts there is a transition part, which transforms particles into a wavefront. A sketch of the framework can be found in Fig. 1 ▶.

In the MC part of our simulation framework, particles are created at the source and transported through the predefined geometry, consisting of different materials. The initial position, direction and energy of the particles created at the source are sampled based on the probability distributions of the implemented source. The simulation of a particle history is terminated when the particle either leaves the geometry or its energy is reduced by scattering to a value below a predefined cut-off energy. In the second case it is assumed that the particle deposits its remaining energy locally and is considered to be absorbed. Within the simulation of the transport of the particle through the geometry, scattering and absorption occur based on probability distributions obtained from the physical scattering and absorption cross sections for the respective material. By simulating a large number of particle histories, precise results can be obtained for the quantities of interest, such as fluence or characteristics of transmitted and scattered particles. In our case, the simulations return the phase space containing variables for location, direction, energy, charge and statistical weight of each particle. The phase space is then transformed into a complex wave amplitude which is passed on to the wave optics part. The wave optics part of our simulation framework models propagation through convolution with a propagator, while phase shifts are obtained through multiplication with transmission functions. The separation of the sample part and the propagation part makes it possible to have a sample-specific but imaging-method-independent sample part and a sample-independent but imaging-method-specific wave optics part. The MC simulation of a sample can therefore be used for different kinds of phase-sensitive imaging techniques such as GI and propagation-based imaging. The different parts are described in more detail below.

Owing to the separation of the imaging method part from the sample part, the scattering which occurs due to the imaging method is neglected. In the case of GI this means that the scattering which occurs in the gratings is neglected. However, for an energy of 25 keV, which was used for GI, the noise introduced by scattering within the grating can be neglected due to the small scattering cross section and the wide scattering angle of Compton and Rayleigh scattering at this energy. To accurately account for the noise, an additional simulation could be run where the gratings are simulated within MC and the intensity resulting from scattering could then be added as noise to the signal of the combination simulation.

#### 2.1.1. MC part   

**Figure 2:**
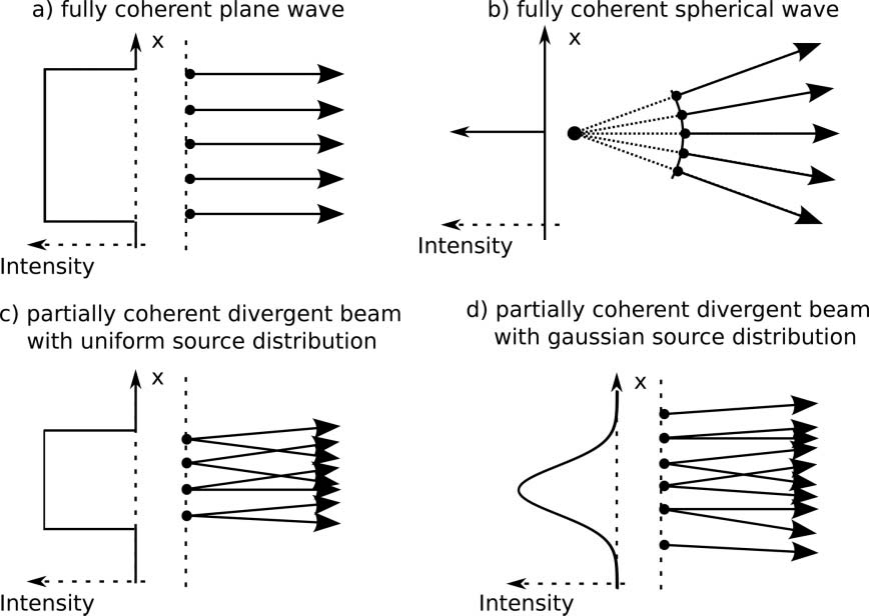
Sketch of implementation of different source types within the framework. (*a*) A fully coherent plane wave, where all particles have the same initial direction and are uniformly sampled over the beam area. (*b*) The situation of a fully coherent spherical wave where all particles start at the same point and the direction is uniformly sampled. (*c*) A partially coherent divergent beam with a uniform distribution of the initial position over the source. (*d*) A partially coherent divergent beam with a Gaussian distribution of the initial positions. The directions in (*c*) and (*d*) are sampled according to the beam divergence.

The MC part of the framework containing the sample and the X-ray source is based on egs++, a c++ implementation of the well established *EGSnrc* code (Kawrakow, 2005 ▶; Kawrakow *et al.*, 2010 ▶). For the considered energy range *EGSnrc* includes Compton and Rayleigh scattering as well as photoelectric absorption. For those scattering events the default *EGSnrc* form factors and cross sections were used (Kawrakow *et al.*, 2010 ▶). For phase-sensitive X-ray imaging it is necessary to take the phase shift of the X-rays passing through a sample into account. Therefore the MC code was extended by introducing the optical path length as an additional variable for each particle. The optical path length is the path integral of the real part of the decrement of the refractive index δ multiplied with the wavenumber *k* and accounts for the phase shift Φ of the particle *p* passing through a material along the path 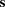,
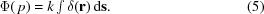
The particle transport occurs stepwise from one part of the geometry or interaction site to the next, thus in each step the path is determined and the respective phase shift is added to the optical path length of the photon. In the MC part, the coherence of the source can be considered through the initial values of the optical path length, energy, direction and position of the photons. A perfectly coherent plane-wave source, for instance, can be obtained if all photons are starting from a plane perpendicular to the beam direction and have the same initial energy, direction and optical path length. Thus at any distance from the source, all particles that did not undergo any interactions will have the same optical path length. Another example would be a perfectly coherent point source, which can be generated by using the same starting point, initial energy and optical path length for all particles, while the initial direction of each particle is sampled uniformly over all directions. To obtain a partially spatially incoherent source within our model, photons can be assigned an initial position and direction or an initial phase, where phase and direction are sampled according to the source distribution that was, for instance, experimentally determined. If the direction for particles starting in one point is sampled according to a distribution, two particles arriving in the same place will not necessarily have originated in the same position. If this is the case, their paths will be different, as will their optical path lengths and with it their phases. Thus the particles will no longer be coherent. Illustrations of some of the possible sources within the model are shown in Fig. 2 ▶. With this, it is possible to account for the effects on the imaging process caused by finite coherence of the source. In principle, it would be possible to also include the electrons generating the photons within the simulation process of the source. This would allow for an intrinsic simulation of the photon source from the electron beam within the model, but it would lead to a simulation cost that is orders of magnitude higher.

In addition to the phase shift, the change of the particle direction due to the refraction process at surfaces was included through a new subroutine. It is called at each transition of a photon from one material into another, similar to the one described by Wang *et al*. (2009*a* ▶). In this subroutine the direction vector of a particle passing from one material with refractive index 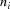 to another material with refractive index 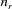 with an entrance angle 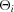 is changed by determining the angle of refraction 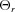 according to Snell’s law (Born & Wolf, 1999 ▶),
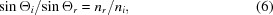
and changing the direction vector of the particle accordingly. The angle of refraction depends only on the incident angle and the refractive indices of the materials. For large angles of incidence with respect to the surface normal, Snell’s law predicts that the sine of the angle of refraction is larger than 1. In this case total external reflection occurs and the photon is reflected at the surface which is also taken into account within the routine. As seen in Fig. 1 ▶, including refraction implies the optical path length to be equal to the actual path integral [see equation (5)] and not to the more intuitive but simple line integral. The latter would be equivalent to the so-called projection approximation (PA), often used in wave optics, which assumes a thin sample, such that the angular deviation due to the refraction is negligible. It has been shown that the projection approximation is valid for large propagation distances compared with the object size (Morgan *et al.*, 2010*a* ▶,*b* ▶); therefore, modelling the change in particle direction due to the refraction may not be relevant for the phase and absorption signal. However, to accurately model the small-angle scattering within the sample, and thus to realistically simulate the dark-field signal, the inclusion of the refraction is essential. Both the refraction subroutine and the optical path length require the input of the real decrements of the refractive indices of all simulated materials. Those can be obtained using equation (4).

#### 2.1.2. The transition from MC to wave optics   

For the wave optics part, the resulting phase space of the MC part needs to be converted into a complex wave amplitude. A complex amplitude is obtained through associating each particle with a wave,
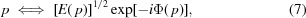
where 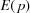 is the energy of the particle and 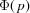 is the optical path length as defined in equation (5). Within the MC simulation, the plane behind the sample is divided into a grid of 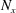 × 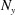 areas 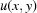 of size 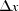 × 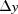. All waves corresponding to particles which fall into the same area are summed up under consideration of their phase. This is repeated for all areas 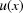 which results in a wavefront 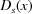 after the sample *s* with
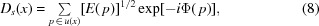
where
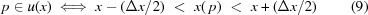
and 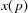 is the position of particle *p* within the area 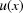. To reduce the computational effort, only one dimension was considered by setting 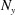 = 1. For a sample which is translation invariant in the *y* direction, this is equivalent to considering only one slice of the tomogram, or one line of the projection image. In the case of a parallel beam set-up, the scattered particles that scatter into the slice from outside the direct projection direction can then be accounted for by setting the size of the source to sufficiently larger than 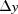. The wavefront 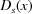 is then passed on to the wave optics part.

#### 2.1.3. Wave optics part   

As previously stated, the wave optics part is adaptable to the specific imaging method. In general, the intensity obtained at the detector 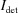 is a function of the amplitude obtained from the transition,
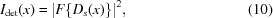
where the function *F* is defined by the imaging method. We will provide the function *F* for two methods in the following section: propagation-based imaging and GI. Due to the combination of MC and wave optics, the number of simulated photons determines the accuracy of the simulated interference pattern but does not correspond to the photon statistics related noise in the final image.

### 2.2. Experimental validation   

#### 2.2.1. Propagation-based imaging   

**Figure 3:**
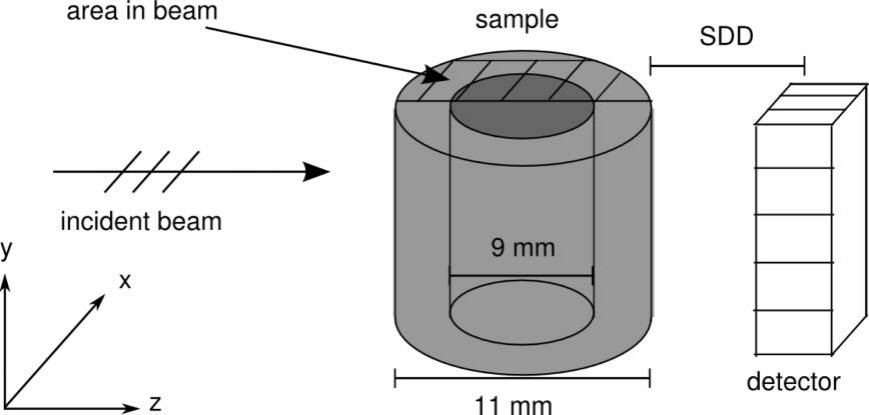
Sketch of the experimental set-up. The source-to-sample distance was 25 m at an energy of 10 keV. The shaded area shows the area of the cylinder within the beam.

In the case of propagation-based imaging, the complex wavefront from the transition of the phase-space file of the MC simulation is propagated to a plane at distance *d* from the sample through convolution with the free-space propagator 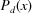,
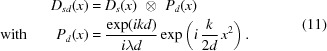
To obtain the intensity of the signal on the detector at distance *d*, the square of the absolute value of 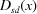 is taken, 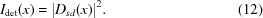
To validate the simulation framework for propagation-based imaging, we compared data simulated with our approach with data obtained with the projection approximation as well as with measured data. The signal of an X-ray beam impinging on a hollow cylinder with outer radius of 5.5 mm and inner radius of 4.5 mm consisting of polypropylene was simulated and measured. A sketch of the experimental set-up is shown in Fig. 3 ▶.

The MC signal was created using the MC part of the framework as described above; the finite source size and small beam divergence were accounted for within the simulation of the source. The PA signal was obtained using the transmission function of the sample. Both signals were propagated to the detector plane through convolution with the free-space propagator using equation (11). The measurements were performed at the TOMCAT beamline (Stampanoni *et al.*, 2006 ▶) at the Swiss Light Source (Villigen, Switzerland) with a source-to-sample distance of 25 m and at a photon energy of 10 keV. Comparisons were made for the following three different sample-to-detector distances (SDD): 1.5 mm, 3 mm and 10 mm.

#### 2.2.2. Grating interferometry   

**Figure 4:**
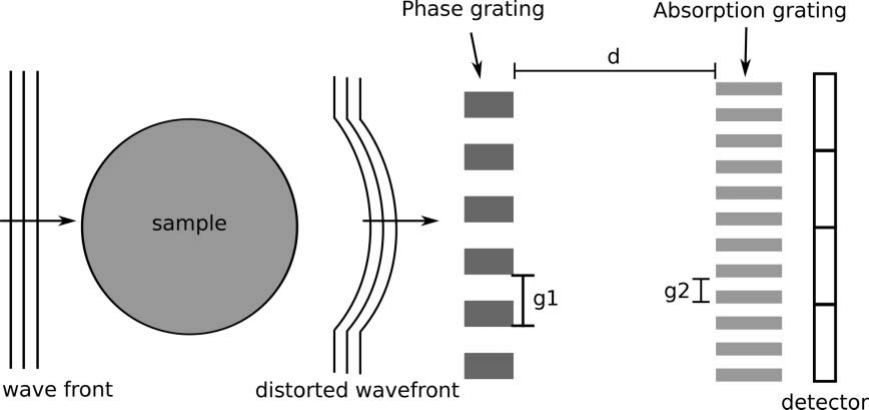
Sketch of the experimentalal set-up of the grating interferometer.

The experimental set-up of a grating interferometer is shown in Fig. 4 ▶. It consists of an X-ray source, a sample and two gratings. The first grating, located right behind the sample, is usually a phase grating with period 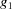, which generates an interference pattern of period 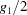 at the so-called Lohmann distances (Suleski, 1997 ▶). For a phase grating with a phase shift of π, the Lohmann distances for a parallel beam are given by
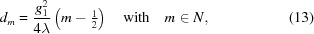
where λ is the wavelength of the X-rays. If a sample is placed between source and grating, the interference pattern is shifted due to the phase shift introduced by the sample. Since the period of the interference pattern is usually smaller than the pixel size, a second grating is required to detect this shift. The second grating is an absorption grating with the same period 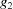 as the interference pattern. By scanning either the first or second grating in multiple steps over the period 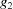, a phase-stepping curve can be obtained for each pixel (Weitkamp *et al.*, 2005 ▶). The shift 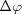 of the interference pattern introduced by the sample is proportional to the refraction angle α through
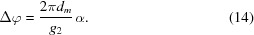
The first derivative of the phase shift Φ of the wavefront introduced by the sample is proportional to the phase shift of the fringes 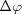 through (Weitkamp *et al.*, 2005 ▶)
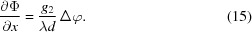
The total phase shift Φ of the sample can then be obtained through integration and the refractive index decrement δ can be calculated using equation (5).

For the simulation of GI, the signal 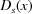, obtained from the MC part of the framework, is propagated through the two gratings by first multiplying the amplitude with the phase-shift function 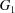 of the phase grating,
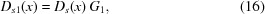
neglecting the absorption of the phase grating. To propagate the signal to the absorption grating located at distance *d* from the phase grating, a convolution with the free-space propagator 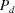 is performed,
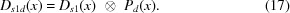
The resulting amplitude is multiplied by the transmission function 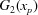 of the absorption grating at position 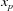, 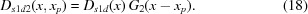
The intensity 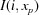 in the detector pixel *i* at phase-step position 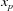 is the integral over the whole area of the pixel of the square of the absolute value of 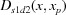,
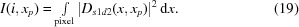
This procedure is repeated for all phase-step positions 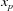. The projection images of the three different contrast modalities are then obtained from the intensity using a Fourier-based approach (Pfeiffer *et al.*, 2006 ▶, 2008 ▶).

For tomography, these steps are repeated for different rotation angles of the sample and the resulting projection images are reconstructed using the reconstruction algorithm *gridrec* (Marone & Stampanoni, 2012 ▶).

To validate the framework for GI, simulations and measurements of two phantoms were compared.

**Figure 5:**
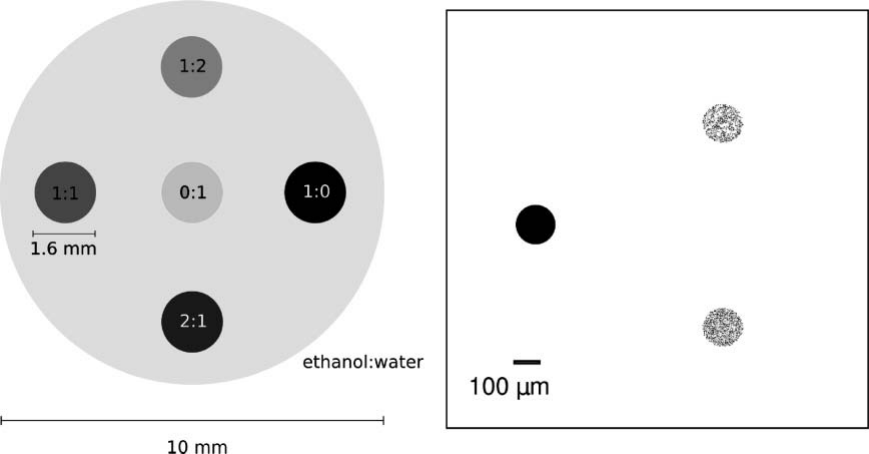
Left: sketch of the phantom for the absorption and phase signal; the innermost cylinder was filled with water, the outer cylinders were filled counter-clockwise with liquids of increasing ethanol concentration. Right: sketch of the second phantom, used to compare the dark-field signal. The cylinder on the left is the full PMMA cylinder, the area on the right on top contains 381 cylinders, and the area at the bottom contains 795 cylinders.

The phantom used for the validation of the absorption and phase-contrast signal consists of a polystyrene (PS) cylinder with five cylindrical holes as shown in Fig. 5 (left) ▶. The holes were filled with five different concentrations of a water and ethanol mixture.

The liquids were pure ethanol, pure water and mixtures of water and ethanol with mass ratio 1:1, 1:2 and 2:1. The theoretical values for δ were calculated using equation (4); the theoretical values for μ were obtained from the NIST database. The theoretically calculated δ and β values for all liquids are shown in Table 1 ▶. To avoid artifacts from high phase-gradients between the sample and surrounding air, the sample was placed in a water-filled aquarium.

The measurements were carried out at the TOMCAT beamline (Stampanoni *et al.*, 2006 ▶) at the Swiss Light Source. 1081 projections over 180° with five phase steps and a pixel size of 7.4 µm were acquired at an energy of 25 keV and with the absorption grating placed at the second Lohmann distance, which is close to optimal imaging conditions (Modregger *et al.*, 2011*a* ▶). Further details about the experimental arrangement can be found by McDonald *et al.* (2009 ▶). For the simulation, the same parameters as for the measurement were chosen. Per projection 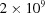 histories were calculated with an energy cut-off of 10 keV. The cut-off was chosen to obtain a certain simulation efficiency and the cut-off level was set under consideration of the mean free path of photons in water, which is 2 mm at 10 keV. To obtain the same degree of coherence in simulation and experiments, the finite source size was modelled by sampling the initial position and direction of the photons within an area with the same second moment as the source at the TOMCAT beamline, which is 53 µm. Since for grating interferometry only the spatial coherence in the horizontal direction, perpendicular to the gratings, is relevant, this was only done for the horizontal direction.

To investigate the effects of small structures, the simulation of a phantom with small substructures is considered. The phantom consists of three circular areas each with a radius of 0.075 mm. The first area contains a full polymethyl methacrylate (PMMA) cylinder, the other two are filled with small PMMA cylinders each with radius of 1 µm. The second area contains 381 of these cylinders; the third contains 795. The PMMA cylinders are distributed randomly within the two areas. A sketch of the phantom is illustrated in Fig. 5 (right) ▶. For these simulations, 541 projections were taken over 180°, the pixel size of the projections was set to 4 µm, the photon energy was set to 25 keV and the absorption grating positioned at the second Lohman distance.

## 3. Results and discussion   

### 3.1. Validation for propagation-based imaging   

**Figure 6:**
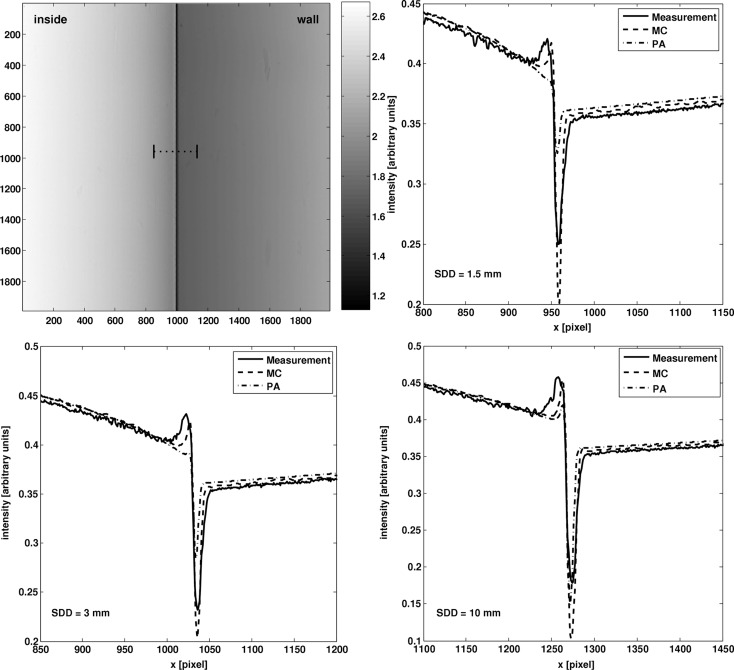
Top left: projection image obtained at 10 keV with a sample-to-detector distance of 3 mm. The dashed line indicates where the profiles for the comparison were taken. Top right to bottom left: comparison of the line profile for MC and projection approximation (PA) to a measurement in a plane 1.5, 3 and 10 mm behind the object.

Fig. 6 ▶ shows the absorption signal of the inner edge of the hollow cylinder as shown in Fig. 3 ▶, for three different sample-to-detector distances.

The images show a comparison of line profiles from the measurement signal, the MC signal and the PA signal. The fringes observed in the experiment are much better approximated by the MC signal than the PA signal for the smaller sample detector distances. For larger sample detector distances, *i.e.* longer propagation distances, the PA signal approaches the MC signal as would be expected. The difference between MC and PA for the small propagation distances is due to the inclusion of the refraction in the particle transport within the sample, which is one of the main aspects of this framework. Due to the refraction, the fringes are already included in the MC signal. However, they are not considered in the PA signal, since there the sample is assumed to be thin and thus angular deflection is neglected. The differences between MC signal and measurement can be explained by uncertainties in the composition, density and surface roughness of the cylinder. The surface roughness, which was not considered in the simulations, has a high influence on the signal, since small substructures in the surface will lead to a broadening of the fringe coming from the inner edge of the cylinder. This is also indicated by the fact that the right fringe is better approximated than the left fringe. This result shows that the refraction is modelled accurately within this framework and thus it can be used for accurate simulations of phase-propagation imaging.

### 3.2. Validation for GI   

**Figure 7:**
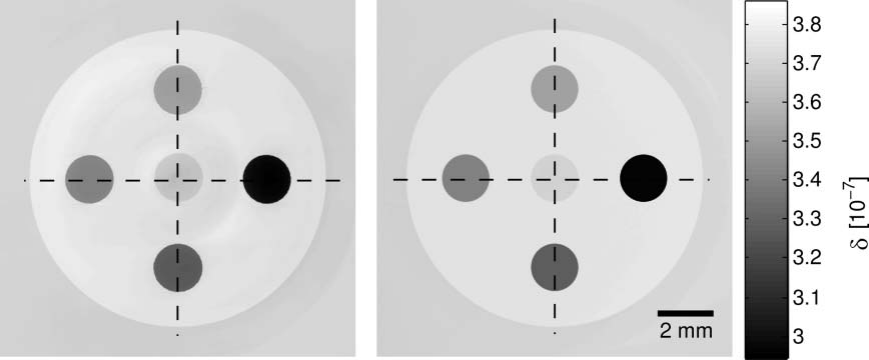
Reconstructed phase image of the measurement (left) and MC simulation (right) of an ethanol–water phantom. The dashed lines indicate the line profiles shown in Fig. 8 ▶.

The reconstructed phase images for simulation and measurement are shown in Fig. 7 ▶. The qualitative agreement of the two images is excellent which shows that the phase signal is simulated in a realistic way. The correlation coefficient for the two images is 0.96 and the normalized mean square error is 0.0006.

**Figure 8:**
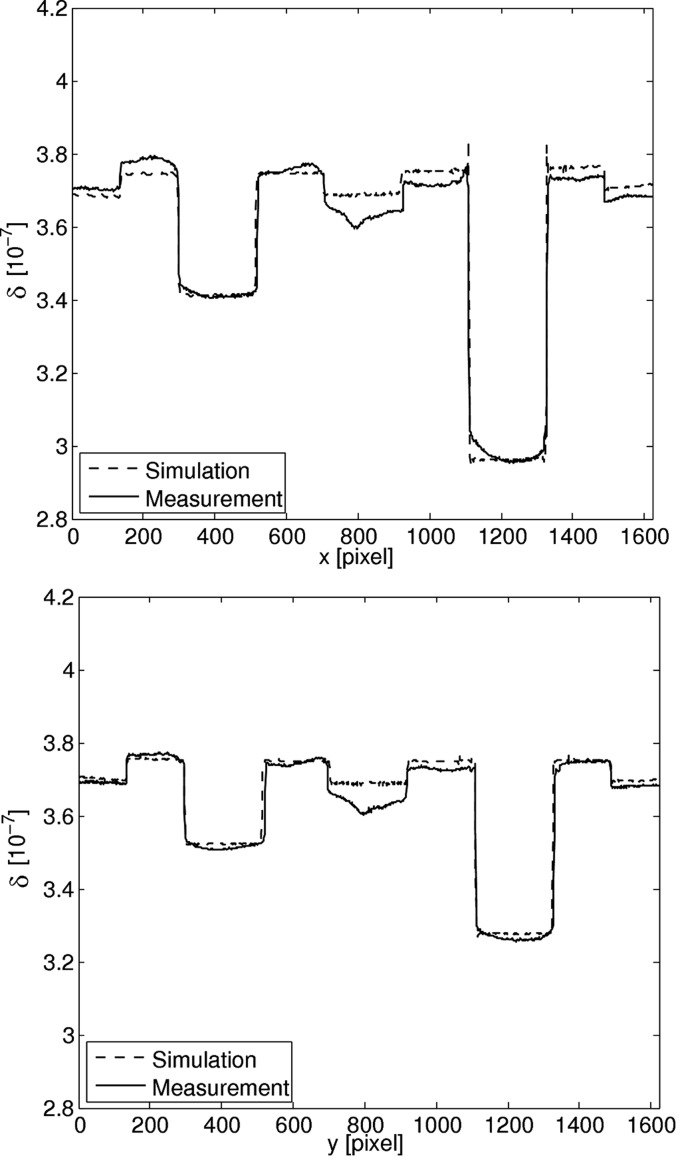
Line profile through the measured and simulated phase image along the horizontal (top) and vertical (bottom) line indicated in Fig. 7 ▶.

In Fig. 8 ▶ the profiles along the two lines shown in Fig. 7 ▶ are depicted. They show that the values agree well for the water–ethanol mixtures and the PS, but not for the water in the middle. There is also a peak artifact visible in the middle. This may be due to water impurities in the measurement. Although Milli-Q water was used in both phantom and the aquarium, contamination cannot be completely excluded. Further possibilities for this effect might be drift of the beam in the experiment, which is a known effect that can only be partially corrected for in the postprocessing. Beam drift was not considered in the simulation. The correlation coefficient for the profiles are 0.99 for the horizontal profiles and 0.98 for the vertical profiles.

**Figure 9:**
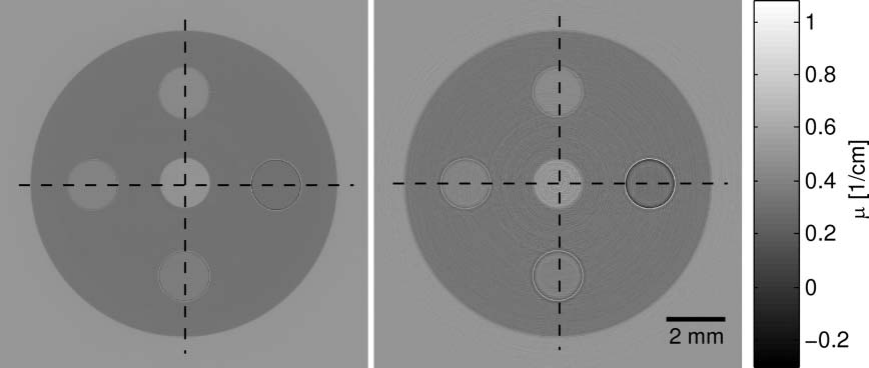
Comparison of absorption measurement (left) and MC simulation (right) of an ethanol–water phantom. The dashed lines indicate the lines along which the profiles were taken.

The reconstructed absorption images for simulation and measurement are shown in Fig. 9 ▶. The two images show a good qualitative agreement; in both absorption images the edge enhancement can clearly be observed. Due to the beam coherence, the edge enhancement occurs at the interfaces of the liquids with high ethanol concentration, which is where the gradient of the phase is largest. It can be seen that the edge enhancement is more emphasized in the simulation than in the measurement, even though the finite source size, which determines the coherence, was considered in the simulation. This is due to the detector response, which would lead to a smoothing of the edge and has not been considered. The simulation image shows ring artifacts which are due to the statistical noise from the MC part which is amplified through the wave propagation. The correlation coefficient for the two images is 0.92 and the normalized mean squared error for the two images is 0.0061.

**Figure 10:**
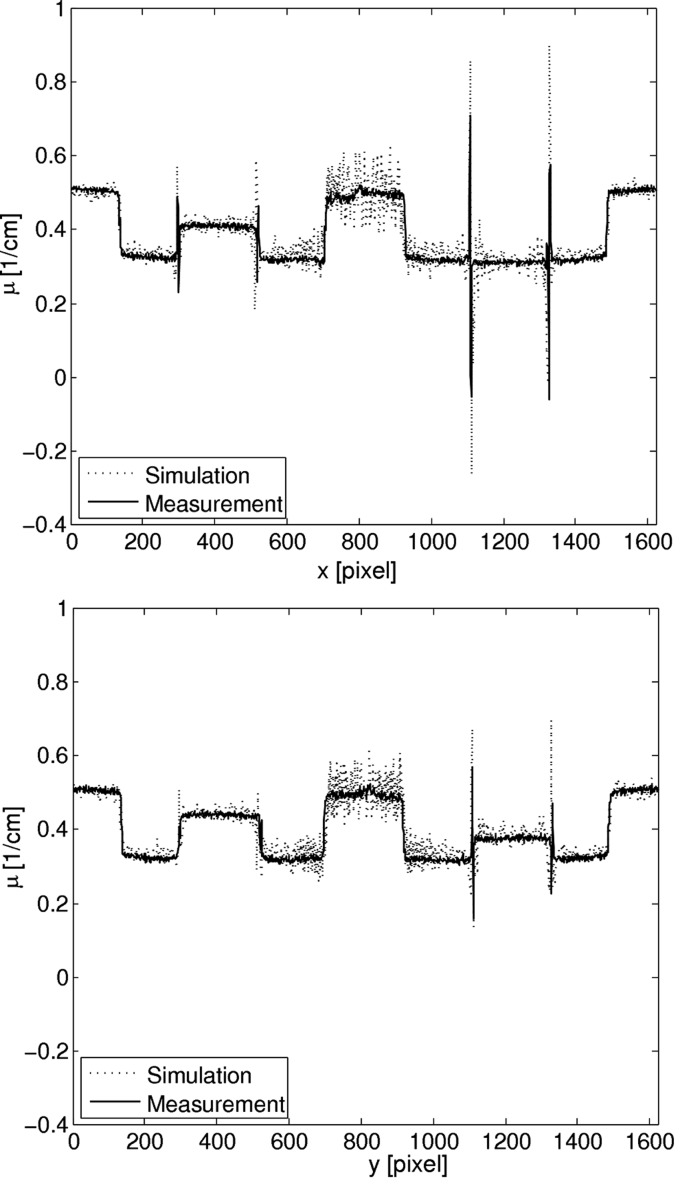
Line profile through the absorption image for measurement and simulation along the horizontal (top) and vertical (bottom) line indicated in Fig. 9 ▶.

Fig. 10 ▶ shows the profiles through the absorption images indicated in Fig. 9 ▶. To reduce the noise the profiles were averaged over ten pixels in the direction perpendicular to the profile. The agreement found is good: the correlation coefficient for the horizontal profiles is 0.85 and 0.89 for the vertical profiles.

**Figure 11:**
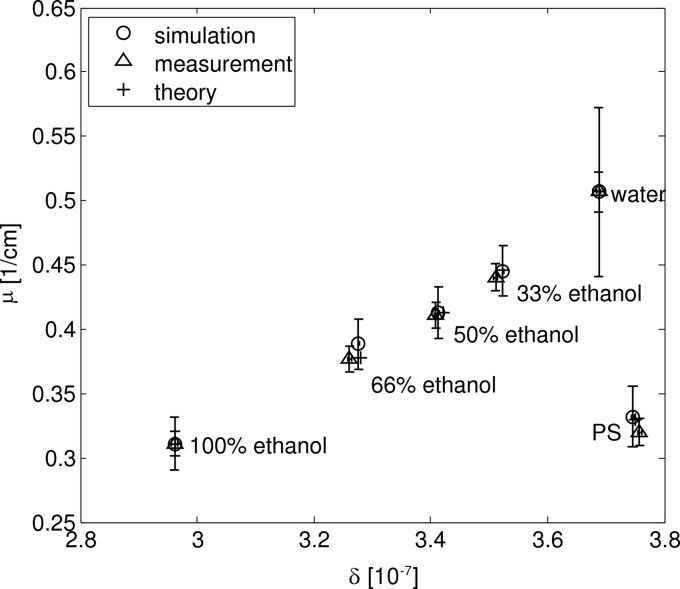
Scatter plot of measured, simulated and theoretical values for δ and μ. The values were calibrated for ethanol and water.

Fig. 11 ▶ shows a scatterplot of the μ and δ values. The values for the δ and μ values were obtained from the reconstructed images by averaging the grey values over several pixels and using equations (15) and (5). To reduce the noise, the measured images were averaged over 30 slices. The values were compared with the theoretical values given in Table 1 ▶. The output of the reconstruction, the measured and simulated values were calibrated to the theoretically calculated values for water and ethanol.

The error bars show the standard deviation of the values within the averaged area. The overall agreement of the values for both μ and δ is very good. The standard deviations for the attenuation coefficients μ are much higher than for the δ values due to the much weaker absorption signal. The differences for the water–ethanol mixtures may be explained by uncertainties in the exact mixture ratio of the two liquids that was measured. The PS cylinder also displays differences between the theoretical and simulated values for μ which may be due to the fact that the exact composition and density of the PS cylinder was unknown, so for the theoretical calculation and the simulation the composition was assumed to be the pure polymer C_8_H_8_ with a density of ρ = 1.04 g cm^−3^, according to values found in the literature. While this results in a good agreement for δ, there is a visible difference in μ, suggesting that these assumptions may not be completely accurate.

**Figure 12:**
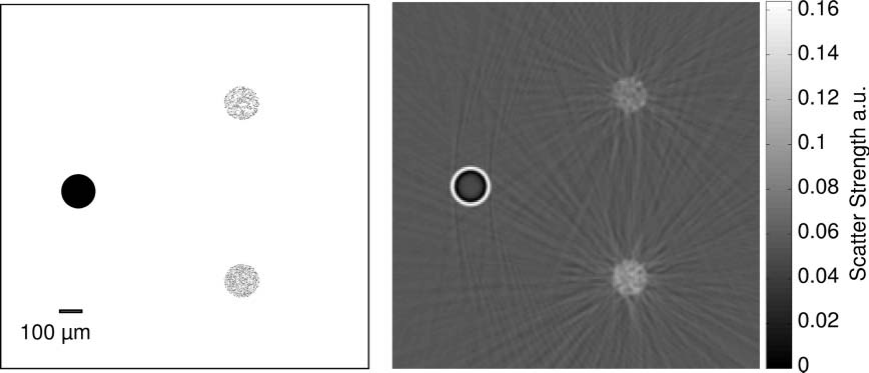
Sketch (left) and reconstructed dark-field images (right) of the artificial sample [the same as Fig. 5 (right) ▶]. The cylinder on the left is the full PMMA cylinder, the area on the right at the top contains 381 cylinders, and the area at the bottom contains 795 cylinders.

The simulated and reconstructed dark-field image of the second sample are shown in Fig. 12 (right) ▶. For comparison, a sketch of the phantom is shown in Fig. 12 (left) ▶. It can be clearly seen that the three areas give a different dark-field signal. As expected, the homogeneous cylinder area yields a dark-field signal only at the edges, while the areas filled with less and more cylinders yield, respectively, a weak and strong dark-field signal from within the whole circular area. The area with the higher number of cylinders gives a stronger signal than the area with fewer cylinders, as would be expected. The observable streak artifacts are most likely due to violations of the basic assumptions about the scattering in the model used for tomographic reconstruction of the dark-field (Modregger *et al.*, 2011*b* ▶). Our results indicate that simulation of the dark-field signal can be obtained qualitatively with our model, provided that all substructures of a sample are known and included in the geometry of the sample. This offers the possibility to obtain a deeper understanding of the dark-field contrast formation process, which is closely related to the scattering and sub-pixel refraction properties of the sample. The framework allows for accessing both the scattering as well as the distributions of refraction directions in one voxel, which can be used for future investigation into the nature of dark-field as well as ultrasmall-angle X-ray scattering.

In general, exact numerical simulation of coherent effects could be achieved by utilizing Huygens’ principle in the MC part of the developed framework. Treating each particle at each spatial step as a new point source by particle splitting, a fully coherent simulation would be accomplished. While this opens the possibility to simulate interference and to include the gratings within the MC part, it would be computationally extremely expensive. The MC part of the model we present in this paper constitutes a first-order approximation to the fully coherent imaging formation process. The results suggest that our approach can be used for the reliable simulation of coherent X-ray imaging.

## 4. Conclusion   

We have developed a framework for the simulation of phase-sensitive X-ray imaging which takes into account both particle- and wave-like properties of the X-rays by combining MC with wave optics simulation. The combination was achieved by splitting the simulation into a sample-dependent MC part and an imaging-method-dependent wave optics part. To take into account coherent effects, the MC part was extended by including refraction and the optical path length.

As a validation of the framework, comparisons between measurements and simulations of a phantom were carried out. A comparison between simulated and measured propagation images was performed which showed that the proposed MC model accounts for the edge enhancement in the simulation of propagation-based imaging. A second comparison was performed for the case of GI where a plastic cylinder phantom with holes filled with different ethanol–water mixtures and a plastic phantom were used. The comparisons showed excellent agreement (correlation coefficient >0.925) between measured, simulated and theoretically calculated values for both the attenuation coefficient μ and decrement of the refractive index δ. This shows that the combination of wave optics with MC was successful and the relevant physical processes were modelled accurately within the simulation. Future applications of this framework could now be investigations into X-ray phase-contrast formation mechanisms through simulation of different sample parameters. Since the framework can also be used for different phase-sensitive X-ray imaging methods, it would also be applicable for the optimization of different set-ups, for instance in investigation of high-energy set-ups for GI.

## Figures and Tables

**Table 1:** Theoretical (A), simulated (B) and measured (C) values for δ and μ at 25 keV The measured and simulated values were calibrated to the theoretically calculated values using the values for ethanol and water.

		δ (10^−7^)			μ (cm^−1^)		
Material	Density (g cm^−3^)	A	B	C	A	B	C
100% water*	0.9982	3.687	3.687 ± 0.01	3.687 ± 0.01	0.507	0.507 ± 0.05	0.507 ± 0.01
33% ethanol	0.9487	3.510	3.523 ± 0.004	3.512 ± 0.003	0.446	0.445 ± 0.01	0.442 ± 0.008
50% ethanol	0.9143	3.400	3.421 ± 0.004	3.410 ± 0.002	0.413	0.413 ± 0.01	0.412 ± 0.007
66% ethanol	0.8749	3.281	3.277 ± 0.003	3.261 ± 0.003	0.378	0.389 ± 0.01	0.377 ± 0.007
100% ethanol*	0.7893	2.960	2.962 ± 0.04	2.962 ± 0.005	0.311	0.311 ± 0.01	0.311 ± 0.007
Polystyrene	0.7893	3.715	3.746 ± 0.005	3.754 ± 0.003	0.330	0.332 ± 0.01	0.316 ± 0.007
